# Open Repair of a True Aneurysm of the Superior Mesenteric Artery

**DOI:** 10.1016/j.jaccas.2025.103914

**Published:** 2025-07-03

**Authors:** Laura Rascio, Nicolò Peluso, Simona Sica, Giovanni Tinelli, Tommaso Donati, Ottavia Borghese, Yamume Tshomba

**Affiliations:** a Unit of Vascular Surgery, Department of Cardiovascular and Pulmonary Sciences, Università Cattolica del Sacro Cuore, Rome, Italy; b Unit of Vascular Surgery, Department of Cardiovascular Sciences, Fondazione Policlinico Universitario A. Gemelli IRCCS, Roma, Italy

**Keywords:** aortomesenteric bypass, autologous graft, great saphenous vein, open repair, superior mesenteric artery aneurysm, visceral aneurysm

## Abstract

**Background:**

Current international guidelines indicate endovascular approach as the gold standard for treatment of superior mesenteric artery aneurysms (SMAAs). However, some cases require open surgery because of anatomical or pathologic constraints.

**Case Summary:**

We report 2 cases of SMAAs treated with open repair at our institution: a 46-year-old man with suspected Ehlers-Danlos syndrome and a thrombosed SMAA who underwent an aortomesenteric bypass using a saphenous vein graft and a 65-year-old man with a dissecting SMAA and rapid aneurysm growth who received an interposition graft with the superficial femoral artery. Both had uneventful recoveries.

**Discussion:**

Despite the advantages of endovascular repair, open surgery is crucial for cases with infection, collagenous diseases, or complex anatomy. A tailored approach optimizes outcomes.

**Take-Home Messages:**

Open surgery remains necessary for complex SMAAs. Alternative arterial grafts, such as the superficial femoral artery, can be valuable substitutes.

## Visual Summary

**Figure:**
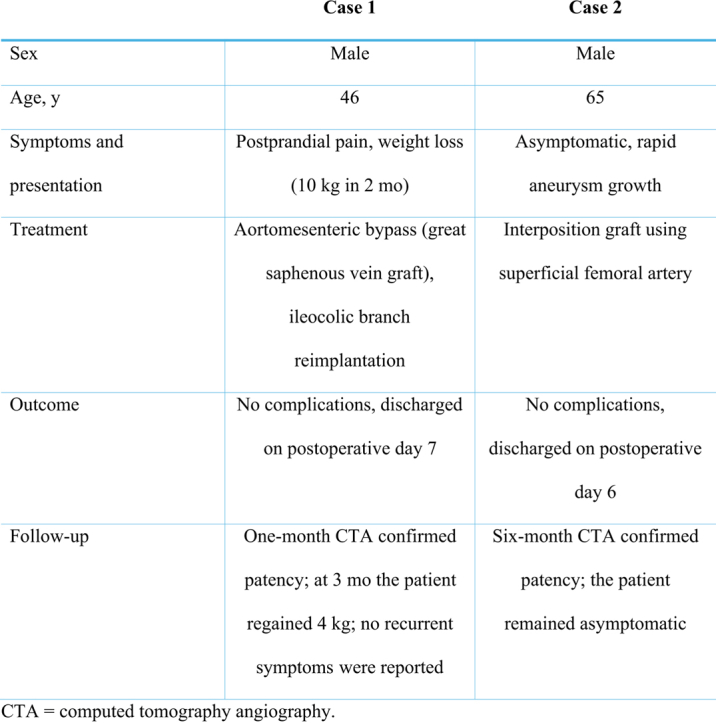

Aneurysms involving the visceral arteries are uncommon (with an incidence of 0.02%^1^) but are still clinically relevant as potentially lethal on rupture. Indeed, mortality rate is reported to be as high as 90% of cases if not promptly treated.^1^^,^^2^ The superior mesenteric artery (SMA) is the site for aneurysm development in up to 15% of cases including both true and false aneurysms.^1^, ^2^, ^3^, ^4^

**Take-Home Messages**

• Open surgery remains necessary for complex SMAAs.
• Alternative arterial grafts, such as the superficial femoral artery, can be valuable substitutes.

Open repair is a safe option for patients who are not eligible for endovascular treatment, as suggested by current international guidelines, particularly those with infections, collagenous diseases, or need to preserve the SMA collateral network.^2^, ^3^, ^4^, ^5^ Herein, we report 2 cases of SMA aneurysm**s** (SMAAs) treated with open surgery at our institution and discuss the advantages of traditional interventions based on individual risk stratification.

## Case Reports

Our institution (Fondazione Policlinico Gemelli IRCCS, Rome, Italy) is a high-volume center for open and endovascular procedures. All cases are discussed in a multidisciplinary team to indicate one approach over the other, balancing the individual risk with the morphology and anatomy of the lesions to be treated. We treat about 30 visceral aneurysms a year (including renal, splenic, and visceral aneurysms), and during the last year, 4 included SMA pathologies. Written informed consent was obtained from the patients for publication of these cases and accompanying images.

### Case 1

#### History of presentation

A 46-year-old man with a suspected diagnosis of Ehlers-Danlos syndrome presented to the emergency department of our hospital with a 3-week history of postprandial pain and recent unexplained weight loss (10 kg in 2 months). He had visited 3 different emergency departments previously without receiving proper diagnosis and adequate treatment for pain relief.

#### Medical history

His medical history included splenectomy for ruptured splenic aneurysm and cholecystectomy for acute cholelithiasis. He has no history of cardiac diseases and denied other relevant medical conditions.

#### Investigations

Computed tomography angiography (CTA) showed a thrombotic occlusion of an SMAA (17 mm) and jejunal, right colic, and ileocolic arteries without signs of bowel ischemia. Because of the anatomical complexity, the lesion was deemed unsuitable for an endovascular approach.

#### Management

After multidisciplinary team discussion, it was decided to perform an aortomesenteric bypass considering the young age of the patient and his comorbidity. Surgery was performed through a standard laparotomy to enter the peritoneum. Ischemic distress of the small bowel was noted in the absence of clear necrotic areas.

**Figure 1:**
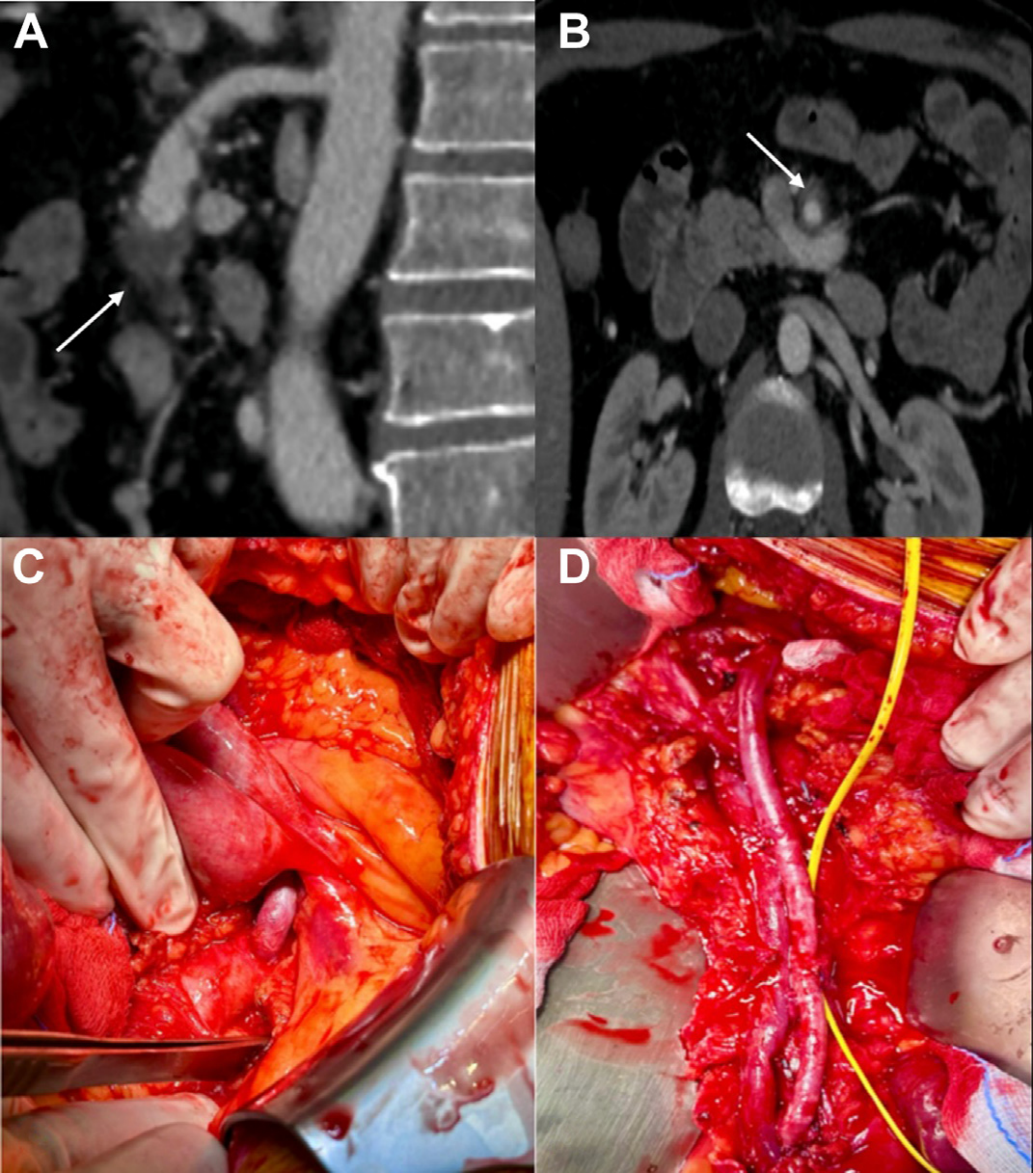
Case 1 – Preoperative Imaging and Surgical Outcome of Aortomesenteric Bypass for Thrombosed SMA Aneurysm Preoperative computed tomography angiography in sagittal (A) and coronal (B) views showing a thrombosed superior mesenteric artery aneurysm. (C) Intraoperative images depicting the proximal anastomosis between the infrarenal aorta and the great saphenous vein graft. (D) Final result: aortomesenteric bypass with distal anastomosis between the great saphenous vein graft and the superior mesenteric artery, along with reimplantation of the first ileocolic branch.

After mobilization of the transverse mesocolon, omentum, and small intestine, the posterior parietal peritoneum was incised and the Treitz ligament was partially dissected. The infrarenal aorta was isolated beneath the left renal vein. The origin of the SMA was then exposed, and the thrombosed aneurysm was dissected proximally and distally (Figure 1). The great saphenous vein was harvested at the thigh, and after systemic heparinization, the infrarenal aorta, SMA, and its collateral branches were clamped. An aortomesenteric bypass was then performed, tunneling the venous graft below the Treitz ligament. Embolectomy of the iliocolic artery was done, and the vessels was reimplanted onto the aortomesenteric bypass. A Bogota bag was placed. After surgery, the patient was transferred to the intensive care unit for close monitoring. On the third postoperative day, he underwent surgical revision, with removal of the Bogota bag and definitive closure of the abdomen.

#### Outcome

The postoperative course was uneventful, and the patient was progressively fed with no recurrence of pain. He was discharged home 7 days thereafter.

**Figure 2:**
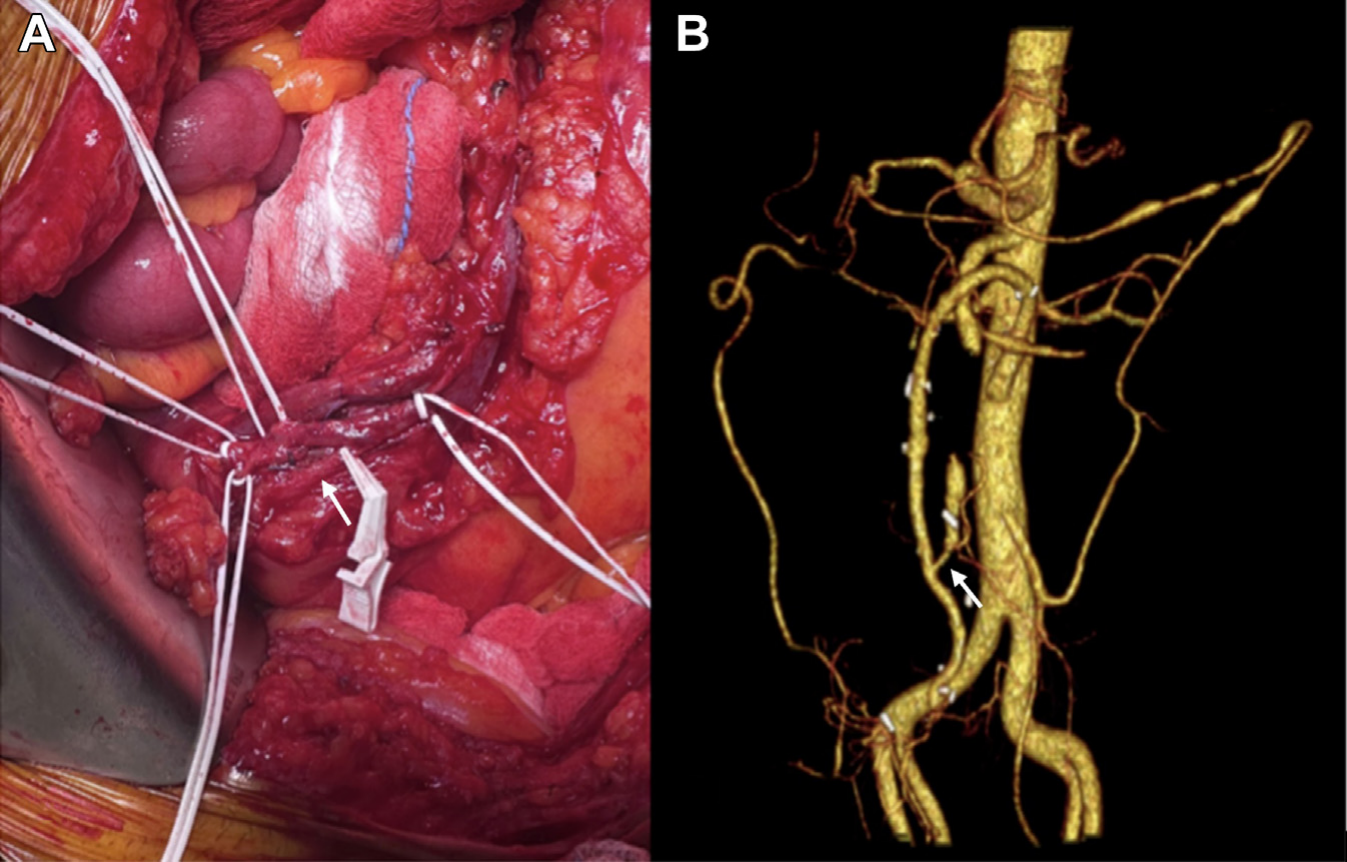
Case 1: Intraoperative and Postoperative Findings of Aortomesenteric Bypass with Ileocolic Branch Reimplantation (A) Intraoperative images showing the aortomesenteric bypass using the great saphenous vein and reimplantation of the first ileocolic branch (arrow). (B) Postoperative computed tomography angiography confirming the patency of the aortomesenteric bypass and the reimplanted branch (arrow).

The 1-month postoperative CTA documented patency of the venous graft and the reimplanted ileocolic branch. Three months postoperatively, the patient was in good clinical condition and reported no postprandial pain. He had gained approximately 4 kg (Figure 2).

### Case 2

#### History of presentation

**Figure 3:**
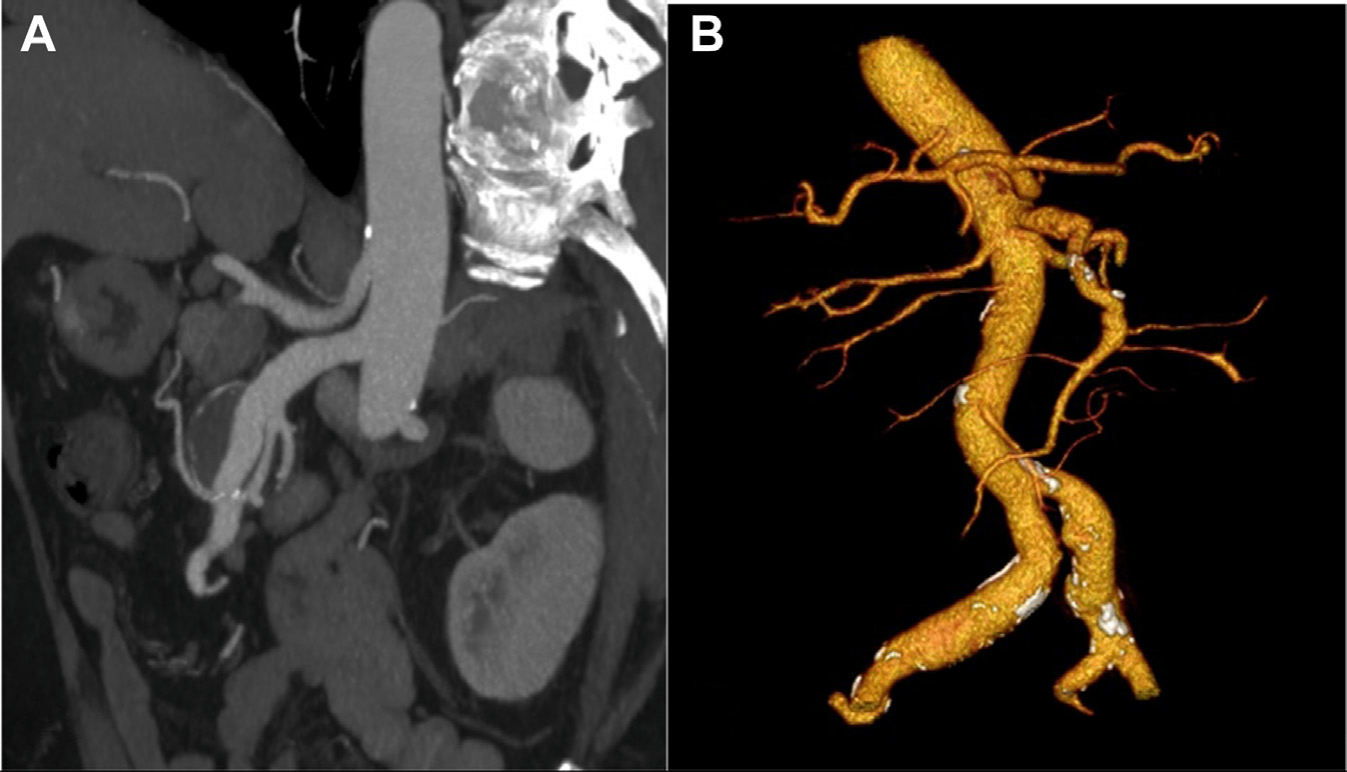
Case 2: Preoperative and Postoperative Imaging of Superior Mesenteric Artery Reconstruction Using Superficial Femoral Artery Graft (A) Preoperative computed tomography angiography showing an aneurysm of the superior mesenteric artery. (B) Postoperative computed tomography volume rendering of the superior mesenteric artery reconstruction with a superficial femoral artery graft.

A 65-year-old overweight man with no history of collagenous disease was at a regular follow-up at our institution for a several-year history of a dissecting SMAA. He presented to our hospital after a rapid increase in the size of the SMAA (29 mm in maximal diameter vs 24 mm 6 months previously) at a follow-up CTA (Figure 3). The patient was asymptomatic.

#### Medical history

The patient had multiple comorbidities and atherosclerotic risk factors, including a history of smoking and dyslipidemia, and multiple prior vascular interventions. Several years earlier, he had undergone a thymectomy. He also had undergone left saphenectomy for venous insufficiency and suffered from myasthenia gravis, requiring medical management. Additionally, he had a long-standing diagnosis of ulcerative colitis and was undergoing treatment for depressive disorder. More recently, he had undergone surgical repair for a left popliteal artery aneurysm

#### Management

The anatomy of the aneurysm did not allow an endovascular repair that could preserve the collateral branches (right colic, ileocolic, jejunal, and ileal arteries) of the SMAA; hence, the patient was scheduled for open repair. With the patient under general anesthesia, a xiphopubic laparotomy was performed. The transverse mesocolon, omentum, and small intestine were mobilized, and through a horizontal incision of the peritoneum at the attachment of the transverse mesocolon, the dissecting SMAA was exposed. Both great saphenous veins were of small caliber, so the left superficial femoral artery was selected as the vascular substitute and replaced with a Dacron bypass. The SMA and its collaterals were clamped, and the superficial femoral artery was used as an interposition graft. The collateral branches were directly reimplanted onto the graft.

#### Outcome

No complications were reported, and the patient was discharged home 6 days after surgery. The 6-month postoperative CTA showed patency of the vascular reconstruction, and the patient was in good clinical condition without any complaints.

## Discussion

Current indications for interventional treatment of SMAAs are a diameter exceeding 25 mm for asymptomatic cases; however, they should all be repaired regardless the size in women of childbearing age.^2^ Currently, endovascular techniques have revolutionized the management of SMAAs, becoming first-line options because of the minimally invasive nature that allows for shorter hospital stays and recovery times and because of the well-recognized efficacy in the short to medium term.^2^, ^3^, ^4^, ^5^ Indeed, endovascular stenting with bare stents alone or with stent-assisted coiling offer safe and effective options in elective settings^2^^,^^4^^,^^5^ and serve as a good alternative in high-risk individuals who could not tolerate general anesthesia or open surgery.^6^ Despite that, open surgical repair (including aneurysmectomy, interposition bypass, endoaneurysmorrhaphy, and ligation^3^^,^^4^) remains pivotal for challenging scenarios where endovascular options are technically infeasible or unsafe.

The main limitations for endovascular repair are infectious aneurysms when aneurysm exclusion should be associated with debridement of infected tissue, whereas the insertion of an endograft could result in further colonization and infection relapse.^7^ Also, collagenous disorders (including Ehlers-Danlos syndrome and fibrodysplasia, etc) are better managed with open strategies by replacing the affected artery with autologous grafts (saphenous vein or arterial substitutes) or prosthesis that will ensure long-lasting results.^8^^,^^9^

Finally, specific anatomical considerations also favor open repair, such as unsuitable distal and proximal landing zone for the endograft, technical feasibility (access to the vessel) or an extensive involvement of collateral branches, and/or an inadequate collateral circulation that would increase the risk of bowel ischemia.^6^^,^^10^ Our institutional experience matches these findings, demonstrating that an individualized approach is critical for optimizing outcomes in complex cases.

## Conclusions

In the treatment of SMAAs, a customized approach based on individual anatomy and risk stratification guarantee the best clinical results. Although endovascular options offer a minimally invasive treatment and are suitable in most cases, open surgery still plays an important role for lesions anatomically unsuitable for endovascular strategies and in treatment of infected aneurysm or collagenous disease.

**Visual Summary:** Overview of Two Cases of Superior Mesenteric Artery Aneurysm Repair

	Case 1	Case 2
Sex	Male	Male
Age, y	46	65
Symptoms and presentation	Postprandial pain, weight loss (10 kg in 2 mo)	Asymptomatic, rapid aneurysm growth
Treatment	Aortomesenteric bypass (great saphenous vein graft), ileocolic branch reimplantation	Interposition graft using superficial femoral artery
Outcome	No complications, discharged on postoperative day 7	No complications, discharged on postoperative day 6
Follow-up	One-month CTA confirmed patency; at 3 mo the patient regained 4 kg; no recurrent symptoms were reported	Six-month CTA confirmed patency; the patient remained asymptomatic

CTA = computed tomography angiography.

## Funding Support and Author Disclosures

The authors have reported that they have no relationships relevant to the contents of this paper to disclose.
